# Mr. Abernethy

**Published:** 1831-06

**Authors:** 


### OBITUARY.

*Death of* MR. ABERNETHY. This distinguished physiologist and surgeon died at his seat at Enfield, on Wednesday, the 20th of April. It is now nearly fifty years ago since Mr. Abernethy became a student at St. Bartholomew's Hospital. The rapid progress which he made in his professional studies soon distinguished him from the crowd of youthful competitors by whom he was surrounded, and the expectations which were hence formed of his subsequent advancement were quickly realised. In his twenty-second year, he was appointed assistant surgeon to the hospital, and soon succeeded Mr. Pott as lecturer on anatomy and surgery. Upon the resignation of Sir James Earle, Mr. Abernethy became surgeon of St. Bartholomew's. In this situation, which offers so many advantages to a man of talent, so many opportunities of improving his profession, Mr. Abernethy greatly distinguished himself as a sound practitioner: his essays on various physiological subjects are well known to the profession; they stamp him as a man of profound and original reflection. In many bold operations of surgery he led the way, and, we believe, he first ventured to put a ligature on the external iliac artery.

Mr. Abernethy ranked higher, perhaps, in the estimation of the public, as a medical, than as a "pure" surgical practitioner; but it cannot be denied that his treatment of general diseases was too confined, too much limited by the opinion that nearly all the ailments of mankind originate in derangement of the digestive organs. It is truly said by a contemporary, that, "perhaps, since the days of Dr. Radcliffe, there has not been a medical practitioner so popular, and so much run after, both for his acknowledged skill, and on account of his eccentric peculiarities: but how contradictory are the reports which are current on this subject. Many of his patients, no doubt, have come away from him affronted, and with their self-importance seriously disturbed; yet how many have still been found anxious once more to try the fate of their reception. What were these, however, to the numbers who visited him, and took their leave, impressed ever after with the strongest sense of his humanity, his superior talent, and in perfect good humour?"

In his public character, Mr. Abernethy was decidedly one of the leading men of his day; and by those who knew him well in his domestic circle, he was highly esteemed for his urbanity and kindness of heart.

